# Editorial

**Published:** 1866-12

**Authors:** 


### Editorial.

CLINICAL INSTRUCTION ON DISEASES OF THE EYE AND EAR. —We are requested to state that the Hospital Committee of the Board of Supervisors have authorized the admission of all medical students to the clinics given in the ward for patients with diseases of the eye and ear, free of charge. The clinics in this department of the County Hospital are given by Dr. HILDRETH, every Saturday morning at 11½ o'clock.

---

CANCER CURERS.—We have recently received a circular from some one styling himself "M. F. BASSETT, M.D.," in which he claims to be able to cure cancerous diseases by the use of some medicine, of the composition of which he acknowledges himself ignorant; yet claims "that no mineral poisons enter into its composition." The excuse plausibly set forth in this circular, for using a secret remedy, will hardly deceive any but the most credulous. If M. F. BASSETT possesses sufficient knowledge to entitle him to place M.D. to his name, why does he not subject the remedy he claims to use to such analysis as will determine its composition? Let neither the profession nor the community be deceived by any such pretenses as are set forth in the circular before us.

---

Chicago Medical Society.—This Society holds its regular meetings on Friday evening of each week. The meetings thus far this season have been well attended. They have been devoted strictly to the consideration of interesting cases, pathological specimens, and the discussion of questions of direct practical importance. At a recent meeting, Dr. J. P. Ross, one of the physicians to the County Hospital, presented several hearts taken from patients who had recently died, showing a very interesting variety of organic or structural changes in that organ. The meeting on the preceding week was occupied with a discussion of the causes and treatment of suppression of urine in connection with attacks of cholera.

---

Close of the Volume.—This number closes the Seventh Volume of the Chicago Medical Examiner. Its publication will be continued the same size and at the same price as at present. As in the past, so in the future, we shall keep two objects prominently in view in the publication of the Examiner. One, to fill its pages with such matter as will be most valuable to the busy, bedside practitioner in the diagnosis and details of treating diseases. The other, to advocate such principles and measures as will improve the educational and social interests of the profession.

---

Prize Questions.—The subscribers, a committee of the Connecticut Medical Society, offer the Jewett Prize, of $200, for the best essay on the following question, *viz.*:—"By what hygienic means may the health of armies be preserved?" And the Russell Prize, of $200, for the best essay on the following question, *viz.*:—"The therapeutic uses and abuses of Quinine and its salts." The offer is extended to all physicians and surgeons of the United States and the British Provinces of North

America. In awarding the prizes, the committee will regard the literary merits as well as the scientific value of the papers submitted; and if no essays are received worthy of such liberal prizes, a decision will be deferred and the time extended. Essays may be forwarded to either of the committee, for the Jewett Prize, on or before the 1st of March, 1867; for the Russell Prize, on or before the 1st of March, 1868. Each essay to be accompanied with a sealed envelope, enclosing the name and address of the author. The unsuccessful essays will remain with the member of the committee in whose hands they were originally placed, subject to the order of their respective authors.

B. H. CATLIN, M.D., West Meriden,
L. J. SANFORD, M.D., New Haven,
HENRY BRONSON, M.D., "

October, 1866. *Prize Committee.*

---

STATISTICS OF COOK COUNTY HOSPITAL.—The results of the first year's operations in Cook County Hospital for the Poor, are shown in the following report to the Board of Supervisors. It must be remembered that Cook County embraces the city of Chicago:—

Supervisor Woodworth presented the report of the medical board of the County Hospital. The following facts are gathered from it:—

Since the organization of the Hospital, in January, 1866, the number of patients under treatment has been 807. Of these, the number admitted into the medical wards has been 505; admitted into the surgical wards, 258; admitted to the eye and ear department, 44. Total, 807.

From the medical wards, the number discharged cured was 326; the number relieved was 50; deaths, 87; remaining, 42. Total, 505.

From the surgical wards, the number discharged after cure, was 208; relieved, 10; deaths, 10; remaining, 30. Total, 258.

From the eye and ear department, there have been discharged after cure, 27; discharged after relief, 5; transferred from the medical department, 1; absconded from the hospital, 3; remaining, 8. Total, 44.

The number of births in the lying-in department, has been 34.

During the month of October, a dispensary for the relief of the outside poor was opened in the basement of the hospital. The number of patients who have applied at the dispensary has been 35. The dispensary is open every day of the week, Sundays excepted, from 1 till 2 o'clock P.M., for medical and surgical cases. Patients with diseases of the eye and ear are admitted at 11 A.M.

The high rate of mortality among the inmates, is owing to that combination of causes which, in public institutions like this, from which the friendless and homeless victims of incurable disease cannot be excluded, always operates to swell the death list beyond the figures which obtain in these hospitals where only acute diseases are treated. Of the deaths which occurred in the medical ward, 45 were cases of chronic, incurable diseases. The recent epidemic of cholera added 8 names to the fatal list. Not less than 20 patients have been admitted in a moribund condition, and have expired within a few hours after their reception.

---

ANNOUNCEMENT OF THE PHYSICIAN'S POCKET RECORD, FOR 30 PATIENTS.—We propose soon to publish from the office of the *Medical and Surgical Reporter*, a new and improved *Physician's Pocket Record*, comprising the following features (subject to such modifications as may be suggested while preparing):—A perpetual calendar; a list of new remedies, their application, doses, etc.; poisons, and their antidotes; treatment of persons asphyxiated; medicinal weights and measures; table for calculating the period of utero-gestation; table for calculating the probable duration of life; classified list of the chief articles of the materia medica, their doses, and market value; table of signs; a visiting list, day-book of accounts and daily memoranda, combined. An Appendix, containing:—An index of patients; obstetric, vaccine, and other engagements, etc.; fee tables, city and country; list of medical periodicals, with their subscription price.

It will be seen that this *Pocket Record* comprises some *new features* that cannot fail to make it the most useful and popular work of the kind in use. It will be neatly, compactly, and substantially got up, of a size more suitable to the side pocket than any now before the profession. It will be so arranged as to be available as a visiting list at any season of the year; that is, it will serve *for a year from the date of purchase.* The visiting list will include a record of visits, a day-book of accounts, and memoranda for each day on the same page.

This *Pocket Record* is intended to meet the wants of both city and country practitioners, and to be an *indispensable companion.* It will not be bulky, although it contains so much material.

As the work is so arranged as to be applicable to any season of the year, we shall issue small editions, so that they can be frequently revised, and any improvements introduced that may be suggested by experience, for we wish to make a *perfect record* for the physician in daily practice. Price $1.50. The *Reporter* and the *Pocket Record* one year, $6.25.

Address, Office of the

MEDICAL AND SURGICAL REPORTER,

*Philadelphia, Pa.*

---

OBITUARY.—SURGEON AND BREVET-BRIGADIER-GENERAL C. S. TRIPLER, U.S.A.—

GENERAL ORDERS, No. 89. } WAR DEPARTMENT, ADJUTANT-GENERAL'S OFFICE, *Washington, Oct. 27, 1866.*

The following notice of the decease of a distinguished officer of the Medical Department of the Army, by the Chief of his Department, is published to the Army:—

"SURGEON-GENERAL'S OFFICE,

"*Washington, Oct. 23, 1866.*

"*To the Adjutant-General, U.S. Army:*—

"SIR:—I have the honor to report the death, at Cincinnati, on the 20th instant, of Brevet-Brigadier-General C. S. Tripler, Surgeon, U.S. Army, Medical Director, Department of the Lakes.

"Entering the Army as Assistant-Surgeon, October, 1830, General Tripler served continuously for thirty-six years, during which time he held, with credit to himself and advantage to the Government, positions of high trust and responsibility, taking part in the Seminole war, the war with Mexico, the occupation of California, and being the first Medical Director of the Army of the Potomac.

"His skilful administration and conscientious discharge of duty has been rewarded by three brevets for 'faithful and meritorious services.' The Medical Corps possesses, in his distinguished career, a bright example of the union of great professional attainments, with the military zeal and pride of an officer, and those qualities which mark the Christian gentleman.

"Very respectfully,

"Your obedient servant,

"J. K. BARNES,

"*Surgeon-General.*"

By order of the Secretary of War:—

E. D. TOWNSEND,

*Assistant-Adjutant-General.*

---

Prize Essays.—We have received, and cheerfully aid in giving publicity to the following liberal offer by Dr. O'Reilly:

New York, 114 West Fourth Street,
(washington square, south,)
November 28, 1866.

JAMES ANDERSON, M.D.,
*President New York Academy of Medicine:*

My Dear Sir—

I send you, herewith, a Bank Book, by which you will perceive I have placed to the credit of the Academy of Medicine six hundred dollars ($600), in the Emigrant Savings Bank, Chambers Street.

I consider that I have fully established the following facts (unfortunately, I regret, highly talented and learned men, who, I presume, have not studied the subject, think otherwise):—

I. That the ganglionic or vital nervous system is the most important organization in man, inasmuch as it is the seat of life.

II. That the oxygen is next in importance, its presence being necessary for the production of the manifestations of life.

III. That the blood ranks next in importance, as it serves for conveying oxygen to the ganglionic or vital nervous tissue, as well as provides for the wear and tear of the body.

IV. That the cerebro-spinal nervous system stands fourth in importance, and is important only as providing for the physical necessities or comforts of man, but not essentially necessary for the preservation of life, as proved by comparative anatomy as well as from facts deducible from other sources.

V. That the ganglionic or vital nerves of the fœtus and the vital or ganglionic nerves of the mother isosculate in the placental lobule, thus establishing a nervous communication between the mother and child.

VI. That life is communicated to the semen much in the same way that electricity is communicated by the torpedo to any object coming in contact with it when that animal is in a state of excitement; or as the pictures of flowers or plants are represented on the bodies of persons killed by lightning, in like manner, the appearance of the internal organization of the male is reflected on the semen at the moment of its emission by the vital shock or current.

I conscientiously believe that no man can scientifically practice medicine or surgery without having a thorough knowledge of the functions of the ganglionic or vital nervous system, the oxygen, and the blood; therefore, that it is the incumbent duty of every lover of the medical and surgical profession to promote the study of these subjects as far as his position enables him to do so. Let any man study the operations of the vital nervous system of the oxygen and the blood, and he will have no difficulty in accounting for the cause of death, the mode in which diseases are propagated and the mode in which they are cured, or why they fail to be cured.

Some time ago, I offered a premium of five hundred dollars for the best essay on the nervous system. As but one essay was handed in, an impartial prize could not be awarded. I would now most respectfully suggest to the Academy the propriety of giving a premium of six hundred dollars for the best "Essay on the Vital or Ganglionic Nervous System, the Oxygen and the Blood, and the Cerebro-Spinal Nervous System," to be competed for by medical students of the United States, and medical graduates of three years' standing.

Physiologists have a very vague idea of the functions of the vital or ganglionic nervous system and the cerebro-spinal nervous system. They confound the functions of both systems together, as if there was only one nervous system, whereas there are two. Hence the confusion, the uncertainty, and the obscurity of their explanations.

With respect to oxygen, all that physiologists seem to know about it is, that if a man has not oxygen he will die; in other words, if a man be drowned, he will be taken up dead; or, in technical language, he will have died of apnœa, or shortness of breath. They don't give a scientific explanation of the cause of death. They appear to be ignorant of the fact that oxygen is the food of life, located in the vital nervous system; that the oxygen is communicated to the vital nervous tissue on the internal coat of the arteries, with which it enters in combination, thus feeding life. They totally ignore the palpable fact that the blood ceases to have any oxygen the moment it ceases to be in contact with the arteries, and consequently with the nerves in the internal coats of the arteries, and the nerves in the capillary extremities of the arteries.

Hoping God will expand the minds of medical men, so as to enable them to unravel and comprehend the operations of the most beautiful, marvelous, and glorious works of His creation, and wishing the Academy a prosperous, a useful, and a scientific career, I have the honor to be, with great respect and esteem,

Your most faithful and obliged servant,

JOHN O'REILLY.

---

GUACO AS A REMEDY FOR CHOLERA.—A series of observations on the treatment of cholera by "guaco," during the worst period of the epidemic at Amiens, France, is presented by Dr. Bourneville, as giving the following results:—1. Confirmed cholera, refractory to ordinary treatment, 10 cases, 6 cures, 4 deaths. 2. Violent cholerine, 4 cases, 4 cures. 3. Choleriform diarrhœa, 4 cases, 4 cures. Mutis, the learned *quinologist*, brought this plant (guaco) to our knowledge, and considered its discovery one of his most valuable scientific conquests. It is unnecessary to say that, concurrently with the cinchonas, guaco was used by the South American Indians long before our *savans* discovered it. For the Indians the bark was the specific for fever, the guaco for poisoned wounds and the like. They used internally the juice of the plant, and externally its powdered leaves. It is but recently, that Dr. Pascal Tardieu and friends made experiments upon guaco prepared in the forms of wine, elixir, and infusion, and found it possessed of valuable properties, arousing to action the entire digestive apparatus.

The *Abeille Medicale*, from which we condense these facts, boasts of the exactness with which the observations were made and recorded, but does not give any details.—*Medical Record.*

---

MORTALITY FOR THE MONTH OF OCTOBER.—Below is given the mortality report for the City of Chicago for the month of October, as drawn from the books in the office of the health-officer of the City. It will be seen that the number of deaths during the month was 1170, an increase over the month preceding of 431, and an increase over the corresponding month of last year of 810, or over three times as many as for the corresponding month of 1865:—

CAUSES OF DEATH.

| | | | |
|---|---|---|---|
| Accidents, | 11 | Fever, Typhoid, | 38 |
| Bronchitis, | 1 | Fever, Typhus, | 1 |
| Cancer, | 2 | Fever, not stated, | 12 |
| Childbed, | 3 | Hemorrhage, | 1 |
| Cholera, | 673 | Hydrocephelus, | 1 |
| Cholera-Morbus, | 15 | Inflammation, | 2 |
| Cholera Infantum, | 9 | Inflammation of Brain, | 12 |
| Congestion of Brain, | 4 | Inflammation of Bowels, | 7 |
| Congestion of Lungs, | 1 | Inflammation of Kidneys, | 5 |
| Colds, | 1 | Inflammation of Lungs, | 3 |
| Consumption, | 43 | Intemperance, | 1 |
| Convulsions, | 46 | Marasmus, | 3 |
| Croup, | 8 | Measles, | 6 |
| Delirium Tremens, | 2 | Neuralgia, | 1 |
| Decline, | 4 | Old Age, | 13 |
| Diarrhœa, | 21 | Poisoning, | 2 |
| Diphtheria, | 12 | Pleurisy, | 2 |
| Disease of Bowels, | 1 | Phthisis Pulmonalis, | 1 |
| Disease of Brain, | 3 | Rheumatism, | 1 |
| Disease of Heart, | 6 | Scrofula, | 2 |
| Disease of Liver, | 4 | Stillborn, | 9 |
| Disease of Lungs, | 6 | Summer Complaint, | 38 |
| Disease of Spine, | 1 | Suicide, | 1 |
| Disease of Throat, | 2 | Teething, | 13 |
| Disease of Hip, | 2 | Tuberculosis, | 2 |
| Dropsy, | 6 | Whooping-Cough, | 19 |
| Drowned, | 4 | Worms, | 1 |
| Dysentery | 10 | Wound, | 1 |
| Erysipelas, | 1 | Uremia, | 1 |
| Fever, Childbed, | 2 | Unknown, | 37 |
| Fever, Remittent, | 17 | | |
| Fever, Scarlet, | 8 | Total, | 1,170 |

Ages of the Deceased.—Under 5 years, 329; over 5 and under 10 years, 95; over 10 and under 20, 66; over 20 and under 30, 202; over 30 and under 40, 190; over 40 and under 50, 116; over 50 and under 60, 72; over 60 and under 70, 47; over 70 and under 80, 22; over 80 and under 90, 5; over 90 and under 100, 1; unknown, 25. Total, 1,170.

NATIVITIES.

| | | | | | |
|---|---|---|---|---|---|
| Chicago, | 331 | Germany, | 223 | Sweden, | 23 |
| Other States, | 260 | Holland, | 2 | Scotland, | 8 |
| Bohemia, | 24 | Ireland, | 168 | Switzerland, | 1 |
| Belgium, | 2 | Norway, | 44 | Wales, | 2 |
| Canada, | 13 | Nova Scotia, | 1 | Unknown, | 20 |
| Denmark, | 3 | On the Sea, | 1 | | |
| England, | 39 | Poland, | 1 | Total, | 1,170 |
| France, | 3 | Spain, | 1 | | |

Mortality for the Month of November.—The following is the mortality report of the Health-Officer, for the month of November, 1866. It presents a marked decrease in the number of deaths from the month of October:—

CAUSES OF DEATH.

| | | | |
|---|---|---|---|
| Accidents, | 16 | Fever, Remittent, | 8 |
| Asthma, | 3 | Fever, Scarlet, | 6 |
| Apoplexy, | 1 | Fever, Spotted, | 1 |
| Burned, | 3 | Fever, Typhoid, | 18 |
| Cancer, | 6 | Fever, not stated, | 4 |
| Childbed, | 4 | Gunshot Wound, | 3 |
| Cholera, | 12 | Hemorrhage, | 3 |
| Cholera-Morbus, | 4 | Hydrocephalus, | 3 |
| Cholera Infantum, | 1 | Inflammation of Brain, | 9 |
| Congestion of Brain, | 6 | Inflammation of Lungs, | 13 |
| Congestion of Lungs, | 4 | Inflammation of Liver, | 1 |
| Consumption, | 39 | Inflammation of Bowels, | 4 |
| Convulsions, | 47 | Intemperance, | 4 |
| Croup, | 11 | Measles, | 1 |
| Cold, | 1 | Marasmus, | 1 |
| Disease of Brain, | 1 | Old Age, | 12 |
| Disease of Heart, | 2 | Phthisis Pulmonalis, | 2 |
| Disease of Liver, | 2 | Paralysis, | 3 |
| Disease of Kidneys, | 1 | Stillborn, | 8 |
| Disease of Hip, | 1 | Summer Complaint, | 5 |
| Disease of Lungs, | 2 | Scald, | 1 |
| Disease of Throat, | 1 | Teething, | 7 |
| Delirium Tremens, | 3 | Tuberculosis, | 2 |
| Decline, | 3 | Uremia, | 1 |
| Dropsy, | 9 | Ulcer, | 1 |
| Diarrhœa, | 5 | Whooping-Cough, | 17 |
| Diphtheria, | 12 | Wounds, | 1 |
| Dysentery, | 2 | Unknown, | 36 |
| Drowned, | 2 | | |
| Erysipelas, | 3 | Total, | 382 |

Ages of the Deceased.—Under 5 years, 173; over 5 and under 10 years, 20; over 10 and under 20, 25; over 20 and under 30, 39; over 30 and under 40, 36; over 4o and under 50, 40; over 50 and under 60, 15; over 60 and under 70, 12; over 70 and under 80, 11; over 80 and under 90, 2; over 90 to 100, 1; unknown, 8. Total, 382.

NATIVITIES.

| | | | | | |
|---|---|---|---|---|---|
| Chicago, | 162 | Norway, | 15 | Switzerland, | 1 |
| Other States, | 72 | Sweden, | 1 | France, | 1 |
| England, | 5 | Canada, | 7 | Italy, | 2 |
| Germany, | 51 | Ireland, | 39 | Unknown, | 18 |
| Denmark, | 1 | Scotland, | 6 | Total, | 382 |

North,......... 73 | South,.........137 | West,.........172 | Total,......... 382

| | |
|---|---|
| Total number during the month of October, | 1170 |
| Total number last year for the month of November, | 299 |
| Decrease from last month, | 788 |

Public Health.—Comparison of Cities.—Dr. Farr, presiding over the late session of the British Science Association, instituted a comparison between the leading nations of Europe, in respect to public health. Russia's death-rate is the highest, if the lecturer's statistics may be trusted, being thirty-six per thousand, while the mean life-time is but twenty-five years, the mortality being greatest in the southern part of the empire.

It would be interesting to know the comparative consumption of brandy in Russia, which is a pretty large item in the undertaker's record.

Italy's death-rate is thirty, and the population of the country is as unhealthy as that of the towns. Rome is the healthiest city on the peninsula, because of her aqueducts. The Germans do not live thirty years on an average, and die at the rate of from twenty-nine to thirty in the thousand. Norway is the most desirable country to live in, since the mean of years is fifty, and the death-rate seventeen. Holland's death-rate is twenty-six. Belgium, France, and England's twenty-two. In England, the mean age is twenty-six, the average length of life, thirty-five. In sixty years, the increase of the Anglo-Saxon race, all over the globe, has equalled the present population of France.

Swinging as a Remedy.—Brown-Séquard recommends the use of the swing as a preventive of nervous paroxysms which recur periodically. In certain cases of hysteria and epilepsy, he has prevented the paroxysm by engaging his patient in violent swinging at the first indicaion of the accession of the fit. The *modus operandi* is easily explained.

